# Work-related experiences and unmet needs of patients with a malignant glioma and relevant professionals: the BrainWork study

**DOI:** 10.1007/s11764-023-01469-z

**Published:** 2023-10-02

**Authors:** Amber Daniëlle Zegers, Pieter Coenen, Amy Heeren, Nadia Takke, Hilko Ardon, Annette Compter, Desiree Dona, Mathilde Kouwenhoven, Sanne B. Schagen, Filip de Vos, Saskia F. A. Duijts

**Affiliations:** 1 Department of Public and Occupational Health, Amsterdam University Medical Centers Location Vrije Universiteit, De Boelelaan 1117, Amsterdam, Netherlands; 2 Societal Participation and Health, Amsterdam Public Health Research Institute, Amsterdam, Netherlands; 3 Musculoskeletal Health, Amsterdam Movement Sciences Research Institute, Amsterdam, Netherlands; 4 Department of Research and Development, Netherlands Comprehensive Cancer Organisation, Utrecht, Netherlands; 5 Department of Neurology, TweeSteden Hospital, Tilburg, Netherlands; 6 Department of Neurology, Netherlands Cancer Institute, Amsterdam, Netherlands; 7 Department of Human Resources, Radboud University Medical Center, Nijmegen, Netherlands; 8 Department of Neurology, Amsterdam University Medical Centers Location Vrije Universiteit, Amsterdam, Netherlands; 9 Cancer Treatment and Quality of Life, Cancer Center Amsterdam, Amsterdam, Netherlands; 10 Division of Psychosocial Research and Epidemiology, Netherlands Cancer Institute, Amsterdam, Netherlands; 11 Department of Medical Oncology, University Medical Center Utrecht, Utrecht, Netherlands; 12 Department of Medical Psychology, Amsterdam University Medical Centers location Vrije Universiteit, Amsterdam, Netherlands

**Keywords:** Glioma, Return to work, Cancer, Patient, Healthcare professional, Qualitative

## Abstract

**Purpose:**

Many patients with a malignant (i.e., grade II-IV) glioma are of working age, yet they are rarely included in “cancer and work” studies. Here, we explored (1) the work-related experiences and unmet needs of patients with a malignant glioma and (2) the experiences and needs of relevant healthcare and occupational (health) professionals (“professionals”) in providing work-related support to this patient group.

**Methods:**

Individual semi-structured interviews were held with patients with a malignant glioma who were of working age and had an employment contract at diagnosis, and relevant professionals. Interviews were transcribed verbatim and analysed thematically.

**Results:**

Patients (*n* = 22) were on average 46 ± 13 years of age (64% male) and diagnosed with a grade II (*n* = 12), III (*n* = 4), or IV glioma (*n* = 6). Professionals (*n* = 16) had on average 15 ± 9 years of relevant work experience with the patient group. Four themes emerged from the data: (1) having a malignant glioma: experienced consequences on work ability, (2) communicating about the consequences of a malignant glioma at work, (3) distilling the right approach: generic or tailored work-related support, and (4) accessibility of work-related support.

**Conclusions:**

Glioma-specific consequences on patients’ work ability necessitate better communication between, and tailored guidance for, patients, relevant professionals, and the workplace. Suggestions for improvement, e.g., the periodic use of comprehensive neuropsychological assessments, are provided in the article.

**Implications for cancer survivors:**

Patients with a malignant glioma would benefit from tailored and proactive outreach about work-related issues bv relevant professionals.

**Supplementary Information:**

The online version contains supplementary material available at 10.1007/s11764-023-01469-z.

## Introduction

Gliomas are primary central nervous system (CNS) tumours developing from glial cells and are the most prevalent type of primary brain tumours in adults [1]. Whilst grade I gliomas have a very slow growth rate and are therefore generally considered benign, grade II–IV gliomas (hereinafter “malignant gliomas”) are increasingly aggressive and related to 2-year survival rates varying between 80% in patients with grade II–III gliomas, to less than 20% in patients with grade IV gliomas [2]. Although the incidence of malignant gliomas increases with advancing age, a substantial number of patients with a malignant glioma is of working age at diagnosis [3]. To illustrate, in the Netherlands, approximately 1200 individuals are diagnosed with a glioma annually; the median age of which is between 46 and 50 years [4]. By intensifying treatment with adjuvant chemo- and radiotherapy post-surgery, the survival rate of patients with a malignant glioma has increased significantly [5], providing some individuals with a choice to continue their working lives.

Work participation can substantially contribute to the financial stability and quality of life of individuals diagnosed with cancer, and of their family caregivers [6–9]. Moreover, work participation can considerably reduce societal costs related to sick leave and productivity loss following cancer [8, 10]. However, patients with a CNS tumour and patients with a non-CNS tumour are 1.78 and 1.37 times more likely, respectively, to become unemployed compared to healthy individuals [11]. These relative risks should not be interpreted to mean that patients with a malignant glioma are generally unable to return to work (RTW). In fact, in a study by Senft and colleagues (2020), 71% of grade II–III glioma patients were able to RTW after surgery and adjuvant treatment [12].

Similar to patients with non-CNS tumours, patients with malignant gliomas can experience work-related barriers, e.g., cancer-related fatigue, and attention and memory disturbances [7, 13–16]. What remains currently understudied, however, are the work-related barriers that might be unique in patients with a malignant glioma. These barriers are likely related to the tumour’s location in the brain, e.g., brain tumour–related epilepsy and cognitive impairments [17], mood disturbances, and personality and behaviour changes [18]. Patients with a malignant glioma are often excluded from studies into work-related experiences of patients with cancer due to (1) the comparative limited amount of patients with a malignant glioma who (are able/want to) RTW, and (2) the hypothesis that the consequences of primary CNS tumours, including gliomas, on patients’ work ability are too unique to be studied within samples of patients with non-CNS tumours. Consequently, a lack of knowledge on the work-related experiences and unmet needs of patients with a malignant glioma currently exists.

Similar to patients who are diagnosed with non-CNS tumours, patients with a malignant glioma can call upon a psychosocial support network of healthcare and occupational (health) professionals (hereafter “professionals”) who are able to provide work-related guidance or referrals, e.g., neuro-oncologists, neurosurgeons, neuropsychologists, occupational therapists, occupational/insurance physicians, and reintegration consultants. Prior research has shown that professionals can experience role ambiguity when confronted with work-related questions in patients with cancer [19, 20]. To date, little is known about the experiences of relevant professionals in providing work-related support to patients with a malignant glioma, what their unmet needs are, and what would help them provide better work-related support to this patient group. Here, we explored (1) the work-related experiences and unmet needs of patients with a malignant glioma, and (2) the experiences and unmet needs of relevant professionals in providing work-related support to this patient group.

## Methods

### Design

This qualitative study was designed and reported based on the consolidated criteria for reporting qualitative research (COREQ) checklist [21] (Supplementary File 1). For information on ethical review and consent to participate, see “Declarations” below.

### Sample and recruitment

Patients were eligible to participate in individual interviews if they (1) were diagnosed with a grade II–IV glioma, (2) were of working age (i.e., between 18 and 66.4 years of age) at diagnosis, (3) had an employment contract at diagnosis, and (4) were able to converse in Dutch unhindered by severe cognitive impairment. Patients were recruited via social media (i.e., Facebook and LinkedIn) and via physicians within three recruiting hospitals. Patients who were recruited via social media were asked to get in touch with the research team and received a study information package after eligibility was confirmed. Patients who were recruited via the hospital received a study information package from their physician after eligibility was confirmed. The study information package contained a patient information letter, an informed consent form, and a short questionnaire indexing essential sociodemographic and medical information (completion time 5–10 min). After receiving the signed informed consent form and completed questionnaire, a member of the research team called the patient to plan the interview date.

Professionals were eligible to participate in an individual interview if they (1) were currently involved in the medical care of patients with a malignant glioma, and/or had experience with providing work-related guidance to this patient group (i.e., providing guidance in RTW, work retention, and/or work cessation), and (2) were able to converse in Dutch. Professionals were recruited via the researchers’ network and via snowball sampling. Eligible professionals were invited for study participation via e-mail and received a study information package, after which the recruitment route was the same as for patients.

Recruitment of patients and professionals continued until data saturation was reached (i.e., no new (sub)themes were identified in the data) and a minimum of 15 patients and 15 professionals were interviewed.

### Data collection

All interviews were conducted from November 2021 until mid-January 2022. Due to COVID-19 restrictions, interviews primarily took place via video call (i.e., using Microsoft Teams/ZOOM.us) or telephone. One interview took place at the patient’s home. Interviews had a mean duration of approximately 1 h and were led by co-authors AH (patients) and NT (professionals). AH and NT were, at the time, MSc students who received qualitative research training prior to conducting the interviews. AH and NT were both present at all interviews to supplement questions and take notes on non-verbal behaviour, unless a scheduling problem necessitated the presence of another co-author (PC or AZ–). SD was present during two interviews to supervise the interview procedure and give suggestions for improvement. PC is a senior researcher in occupational epidemiology. AZ and SD are, respectively, a PhD candidate, and senior researcher in psychosocial oncology. PC, AZ, and SD have prior experience in conducting and reporting qualitative research (e.g., [22, 23]). Occasionally, a patient’s partner was present during the interview. As partners were not asked to participate nor signed informed consent, any remarks made by partners during the interview were not included in this article. No other individuals were present at the interviews. No relationship was established between the patient interviewees and the researchers prior to study commencement. Some professionals were known to the research team prior to study commencement.

Interviews were semi-structured and followed patient and professional interview guides (Table 1), both of which were constructed on the basis of previous scientific literature on the work-related experiences of patients with CNS tumours and other malignant tumours (e.g., [7, 12–15]) and discussions with relevant stakeholders (e.g., a bereaved partner, a neurologist, and a nurse specialist) and the research team. Interview guides were pilot-tested with a patient diagnosed with a malignant glioma, a bereaved partner, and a neurologist, none of whom participated in the study. All interviews were audio-recorded and transcribed full verbatim by AH, NT, AZ, and two research assistants. To protect confidentiality, identifiable information was redacted. No repeat interviews were held and transcripts were not returned to interviewees.

**Table 1:** Abbreviated semi-structured interview guides

Patient interview guide	Professional interview guide
Experiences with work resumption, work retention, or work cessation since malignant glioma diagnosis	Communication timing and style regarding work resumption, work retention, or work cessation with patients with malignant glioma
Glioma-specific symptoms affecting work ability	Extent of knowledge about consequences of malignant glioma on work ability/availability and accessibility of information for professionals on this topic
Communication with workplace, healthcare and occupational (health) professionals about glioma and work resumption, work retention, or work cessation	Extent of knowledge of laws and regulations concerning work resumption, work retention, and work cessation in patients with malignant glioma
(Changed) Importance of work participation since malignant glioma diagnosis	Experiences concerning illness perception and motivation to resume or retain work in patients with malignant glioma
Financial concerns since malignant glioma diagnosis	
Extent to which patients felt informed of laws and regulations concerning work resumption, work retention, or work cessation in light of malignant glioma diagnosis	

### Analysis

Interview data were analysed using a semantic approach, i.e., themes were identified in the explicit meanings of the data. Thematic analysis was conducted on the basis of the six-step framework of Braun and Clarke, i.e., (1) familiarizing oneself with the data, (2) generating initial codes, (3) searching for themes, (4) reviewing themes, (5) defining and naming themes, and (6) producing the final report [24], using ATLAS.ti 9 for Windows (ATLAS.ti Scientific Software Development GmbH). Themes were data-driven. To assess inter-coder reliability, one patient transcript and one professional transcript was independently co-coded by authors AH and NT, after which a consensus meeting was held to discuss and resolve coding discrepancies. The constant comparative method was used to compare codes within and between interviews to identify main themes. Main and sub-themes were discussed within the research team (AH, NT, AZ, SD, PC) until consensus about the interpretation of key themes was reached. Interviewee quotations were selected from the data to support thematic discussions below. Quotations were translated from Dutch to English by the research team. Interviewees did not provide feedback on the findings of the study, but did receive a newsletter detailing study findings on group level. Questionnaire data were analysed with descriptive statistical analyses using Microsoft Excel.

## Results

### Sample characteristics

Of 35 patients who expressed interest in study participation, 22 were eligible and participated in the interviews. Patients who were ineligible had grade I gliomas, did not have an employment contract at diagnosis, or were self-employed at diagnosis. Patients had a mean age of 46 ± 13 years and 14 were male. Patients were diagnosed with a grade II (*n* = 12), III *(n* = 4), or IV (*n* = 6) glioma, respectively, on average 4 ± 3 years ago (range 5 months to approximately 10 years). Most patients *(n* = 13) had a high educational background, e.g., had completed a university degree. Others (*n* = 9) with a low or middle educational background had completed, e.g., lower or upper secondary education, respectively.

Sixteen professionals participated in the interviews. Professionals had on average 15 ± 9 years of relevant work experience and had various occupations, including, e.g., neuro-oncologist, nurse specialist, rehabilitation physician, occupational therapist, medical social worker, occupational physician specialized in oncology, and insurance physician. Respondent characteristics are shown in Table 2. The following themes were identified in the data.

**Table 2:** Characteristics of study population (patients and professionals)

Patient characteristics (*n* = 22)	
Age in years, mean (SD)	46 [13]
Years post-diagnosis, mean (SD)	4 [3]
Sex (*n*)	
Male	14
Female	8
Glioma grade (*n*)	
II	12
III	4
IV	6
Treatment received (*n*)^a^	
Surgery	21
Radiotherapy	20
Chemotherapy	18
Highest educational level^b^ (*n*)	
High	13
Low/middle	9
Work situation at diagnosis (*n*)	
Full-time	15
Part-time	7
Work situation at interview (*n*)	
Full-time	3
Part-time	7
Full work disability benefits or other^c^	12
Professional characteristics (*n* = 16)	
Age in years, mean (SD)	49 [9]
Years of relevant experience with patient group, mean (SD)	15 [9]
Occupation	
Clinical neuro-oncologist	1
Clinical neuropsychologist	1
Hospital-based occupational physician	1
Director of re-integration consultancy	1
Employer of a glioma patient	1
Insurance physician	1
Internist-oncologist	1
Legal advisor	1
Medical social worker	1
Nurse specialist	2
Occupational physician specialized in oncology	2
Occupational therapist	1
Labour expert	1
Rehabilitation physician	1

^a^Respondents were able to select multiple answers

^b^Educational levels: low = ISCED 0, 1, 2; medium = ISCED 3, 4; high = ISCED 5, 6, 7, 8 [52]

^c^Situations in which patients were partly employed and partly on work disability benefits, but contract type was unspecified (full-time/part-time)

#### Theme 1: Having a malignant glioma: experienced consequences on work ability

##### Patients

Some patients were on sick leave pre-diagnosis due to (suspected) burnout or another medical reason. Nearly all patients were diagnosed with a grade II–IV glioma after experiencing an acute epileptic seizure. In some cases, the workplace was witness to the epileptic seizure. Upon the seizure, patients were taken to the hospital, after which subsequent scans confirmed the presence of a malignant glioma. Most patients described a sense of bewilderment and fear due to feeling relatively normal, yet also feeling threatened by the impalpable tumour within their brain and the consequences it could have in terms of epileptic seizures, personality and behavioural changes, and life expectancy.

*"I didn’t notice any tumour-related symptoms. (…) It’s very scary, not knowing what’s happening in your head."* Female, grade II glioma

Most patients reported a high level of fatigue and changes in cognitive functioning post-diagnosis and/or post-treatment. Self-reported changes in cognitive functioning included, e.g., aphasia, memory and concentration impairments, not being able to multitask, and a high sensitivity to stimuli such as noise. Furthermore, some patients reported heightened emotional responsivity, including feeling fearful, anxious, emotionally overwhelmed, and/or irritable. Patients felt the abovementioned symptoms had varying impacts on their work ability. Some said symptoms hindered their RTW directly (e.g., cognitive difficulties making client contact unfeasible; losing one’s driver’s license), or indirectly (e.g., invoking a sense of discomfort in colleagues due to the glioma, see below), and others said symptoms did not impact their work ability noticeably.

*"I sleep about a third more than I did before, I can’t do maths anymore, and I’m more emotional. (…) My problem [also] lies with organisational tasks. If I get a message from my wife at four o’clock saying that she’s away from home, and I have singing class at eight, and in between we need to have dinner, I get confused and become irritable towards her."* Male, grade III glioma

Struggling with RTW or work retention made some patients feel frustrated or ashamed. Patients who decided to stop working post-diagnosis and/or -treatment reported varying emotions regarding work cessation. Some felt relieved because RTW was experienced as unfeasible, whilst others grieved the sudden loss of their occupation as a source of purpose and joy.

*"[At work] other people are dependent on you. And you’re just not a dependable employee anymore."* Male, grade IV glioma

##### Professionals

Regarding patients’ work ability, professionals explained that the tumour’s location was likely more impactful than its grade. Frontal lobe tumours were thought to be especially negatively impactful on patients’ work ability, as gliomas in this location could lead to, e.g., cognitive impairments, reduced illness understanding, and personality and behavioural changes. Professionals stated that some symptoms (e.g., cognitive impairment, sensitivity to stimuli), though also present in patients with non-CNS tumours, are more pronounced in patients with a malignant glioma. According to professionals, in patients with CNS tumours, symptoms are largely due to the effects of the tumour and its location, i.e., acquired brain injury, whereas in patients with non-CNS tumours, symptoms are largely due to treatment (e.g., chemotherapy) and the psychological impact of having cancer. Moreover, whereas an improvement in symptoms and work ability over time is often seen in patients with non-CNS tumours (depending on, amongst others, the grade), professionals stated that for patients with a malignant glioma, symptoms and work ability typically worsen over time.

*"I think that if it [the glioma] is frontal, you’re less likely to return to the same job function [as before the diagnosis]."* Rehabilitation physician

Professionals further described that employers and colleagues of patients with a malignant glioma appeared to find it more challenging to respond adequately to a glioma diagnosis than to other cancer diagnoses. The unpredictable progression of the illness and its sometimes covert physical, cognitive, and psychosocial consequences were said to impact supervisors’ and colleagues’ behaviour towards the patient. According to professionals, supervisors and colleagues felt uncomfortable at times; they were alert for and sometimes afraid of, e.g., epileptic seizures, possible changes in personality and behaviour, and the possibility of the patient passing away. Professionals described that supervisors and colleagues struggled with relying on the patient to do the work, because it was unclear what they could reasonably expect of them in terms of work ability.

*"When it concerns brain tumours, (…) people tend to question the patient’s psychological state, mood, and personality, and whether that has been permanently influenced by the fact that someone has been treated in the head."* Director reintegration consultancy

#### Theme 2: Communicating about the consequences of a malignant glioma at work

##### Patients

Patients stated that they were generally open about the malignant glioma diagnosis towards their supervisor, colleagues, and occupational physician. Occasionally, patients explicitly asked their supervisor and/or colleagues to inform them of observed changes in their work ability, personality, or behaviour, as they were unsure whether they would notice such changes themselves.

*"I gave my colleagues permission to tell me if they noticed anything [unusual about my behaviour]. Because (…) if I’m unaware of it, then I can’t talk about it with my oncologist or neurologist either."* Female, grade IV glioma

Concerning communication about (possible) consequences of a malignant glioma at work, patients had varying experiences with their supervisors and occupational physicians. If the patient experienced the professional relationship with their supervisor satisfactory pre-diagnosis, often, communication about the glioma diagnosis and mutual work-related expectations post-diagnosis was satisfactory as well. Some patients said that their supervisors were hard to reach, or that they faced a lack of interest, compassion, or cooperation from their supervisors, resulting in frustration when patients wished to RTW.

Furthermore, not all patients were aware of the occupational physician’s role in the illness and RTW trajectory, nor did all patients meet with an occupational physician at appropriate (i.e., legally determined) times during this trajectory. When patients met with a professional, some patients felt that they were not being understood or taken seriously, due to a lack of glioma-specific knowledge in the professional.

*"The labour expert really didn’t understand what was going on [with my diagnosis]. (…) He didn’t look into it. (…) When we [my wife and I] read the labour expert’s assessment report, we thought ‘This doesn’t make sense at all. This isn’t me.’ My employer agreed with us. Together with my employer, we wrote a letter stating that we disagreed with the assessment report and requested a second opinion. (…). I don’t know what it would’ve been like, had I been alone in this."* Male, grade IV glioma

Patients who applied for a new job did not always discuss the malignant glioma diagnosis during the interview process. The impalpable and progressive nature of the illness made it difficult for some patients to align their perspectives on their own work ability, with perspectives of prospective employers.

*"When you look at me, you can’t tell [that I have a grade II glioma]. You don’t notice anything [about my appearance or behaviour]. So if I talk about it [having a glioma], then maybe it comes across as disingenuous [during a job interview]."* Female, grade II glioma

##### Professionals

Professionals, such as reintegration consultants, mentioned that their services were often called upon rather late in the RTW or work retention process of patients with a malignant glioma. Often, work-related conflicts already existed. According to some professionals, this delay can be due to limited illness understanding, related to cognitive impairment and/or behavioural changes, in some patients with a malignant glioma. To help patients during their RTW and work retention process, professionals suggested implementing periodic meetings with, e.g., a supervisor or occupational physician, to prevent potential conflict situations. Additionally, as malignant gliomas and the treatment thereof can have consequences in terms of fatigue and cognitive impairment, it was mentioned that professionals can help patients by initiating conversations about work earlier on.

*"We ended up (…) making proper work-related agreements: ‘What [kind of work] are you allowed to do? What [kind of work] are you not allowed to do?’ (…) We put a lot of time into it, had weekly or biweekly consultations on how things were going and what [he] was working on."* Employer

#### Theme 3: Distilling the right approach: generic or tailored work-related support

##### Patients

Bewilderment about the sudden presentation of the illness, mixed with varying degrees of (understanding of) its impact, caused patients to have questions about their current and future work ability. Although compassion was readily available, patients typically did not receive tailored work-related support in- or outside the hospital setting. Patients stated that it would have been helpful if hospital-based professionals initiated conversations about work and communicated clearly with them about the realities and possibilities of working with a grade II–IV glioma.

*"The physician thought it was odd [that I wanted to RTW], so he didn’t really ask about it."* Male, grade IV glioma

*"I’ve often cried about work-related issues at the neuro-oncology clinical nurse specialist. (…) They really care about you [in the hospital], but they can’t help you."* Female, grade II glioma

When considering RTW, patients’ motivations to RTW varied, e.g., financial security, societal and familial responsibility, and/or maintaining a sense of purpose and normality. Patients who wished to RTW explained that they often needed tailored support and personalized workplace adjustments to do so successfully, e.g., altering job responsibilities and tasks, reducing working hours, and adjusting the work environment to prevent sensory overload. When patients received this type of tailored support, e.g., from their supervisor or occupational physician, some were able to gradually increase their working hours. Not all jobs, nor all medical circumstances (e.g., grade, tumour location), were amenable to such adjustments, causing some patients to cease working or attempt to seek work elsewhere.

*"If I had a different job, then I would very carefully try to perform some of my job tasks again. But that’s unfeasible in my job. (…) I found multitasking very easy before, but that’s too difficult now. (…) I don’t think work is feasible anymore, at least not in this job."* Female, grade IV glioma

*"My prognosis is very poor, so [mine] is not a reintegration story. (…) But we have another employee with a brain tumour within the organization, (…) and her symptoms are very different [i.e., worse] from mine, even though her prognosis is more favourable. She’s not back at work either."* Female, grade IV glioma

Most patients stated that when professionals, such as occupational physicians, gave general RTW advice (i.e., “Just do what you can”), it was experienced as unhelpful in their RTW success. Instead of focusing on their personal wishes and individual work-related capabilities, patients said that professionals often focused on the incurability of the grade II–IV glioma, and de facto advised to stop working.

*"My former supervisor pressured me into applying for full work disability benefits. (…) Even the occupational physician said: ‘Man, just enjoy what you have left and stop working.'"* Male, grade II glioma

*"The HR advisor said ‘If you can’t be in front of the classroom, you can’t be a teacher. We don’t have anything else for you*.'*"* Female, grade II glioma

*"My tumour type grows very slowly, and I can basically still do everything [that I used to do pre-diagnosis]. Immediately [upon diagnosis] it’s like: ‘You’re ill, so you can’t work anymore’. People [professionals] don’t include you in finding solutions." *Female, grade II glioma

To address the discomfort experienced by supervisors and colleagues (see Theme 1), patients mentioned that it would be beneficial to provide the work environment with support as well.

*"It was a horrible message to have to share: this is my diagnosis, and it’s obviously not going to get better. I told my colleagues, and they received professional help to deal with it."* Male, grade II glioma

##### Professionals

Professionals stated that patients with a malignant glioma could experience similar difficulties regarding RTW as patients with non-CNS tumours, e.g., fatigue, cognitive impairment, and lack of empathy or support from the employer. Therefore, professionals typically translated their experiences with patients with non-CNS tumours to patients with malignant gliomas.

*"* *It’s not that I have a different approach for that [reintegration of glioma patients]."* Legal advisor

However, the impact of a malignant glioma on work ability described in Theme 1 did necessitate professionals to tailor their work-related guidance, e.g., in cases of frontal lobe–acquired brain injury. Professionals stressed that patients with a malignant glioma would benefit from a neuropsychological assessment to determine the extent and severity of the damage, and that the outcomes of this assessment would be useful for professionals to provide tailored advice for patients regarding RTW, work retention, and work cessation.

Professionals such as occupational physicians stated that, if they were to be better informed about the medical details of patients with a malignant glioma (e.g., received relevant medical data), they would be better able to assess patients’ work ability and therefore tailor their advice. A caveat to obtaining relevant medical data is that, according to Dutch legislation, patients have to provide explicit permission for occupational physicians to (1) access their medical data, and (2) to communicate about their medical data with other professionals. Often, patients were said to feel mistrustful towards occupational physicians, not believing them to act in their best interest nor to act independently from the employer. Additionally, it was said that occupational physicians often did not receive relevant medical data upon request, but rather copies of letters to GPs, in which very little relevant information was provided, hindering adequate assessment and tailoring of advice.

*"I notice that patients sometimes find it difficult to give their occupational physician permission to request all [relevant medical] information. (…) I always advise [the patient] to give permission, because the occupational physician then also has the correct medical data (…) and can then give sound advice [regarding RTW, work retention, or work cessation]."* Clinical nurse specialist

#### Theme 4: Accessibility of work-related support

##### Patients

Most patients received information and advice regarding Dutch laws and regulations concerning, e.g., sick leave, work disability, and reintegration, from their occupational physician and/or human resource manager, and sometimes even from relatives. Despite this, some patients remarked that they missed a clear overview of Dutch laws and regulations concerning sick leave, work disability, and reintegration, necessitating them to piece together bits of information themselves. Laws and regulations were often experienced as rigid and ill-fitting to the illness and RTW journey.

*"I understand that it’s the law, but exceptions should be made for people with cancer. (…) You’re being pushed to RTW when you’re still undergoing treatment. It was horrible. I felt trapped."* Female, grade II glioma

Many patients looked for tailored work-related support, in order to make decisions about whether to RTW, how to RTW, and how to adjust their work, by calling on, e.g., a social worker, psychologist, occupational therapist, or reintegration consultant. Moreover, some patients obtained legal and/or financial advice, e.g., to get informed on their rights and obligations regarding RTW/work cessation, and to ensure the financial safety of their family members upon them becoming work disabled in the future, or upon their eventual passing.

*"I have two young kids,* *so I want to earn a good salary to pay for everything. But I don’t know if I can still* *do that 10 to 15 years from now.* *And I worry about that. I know that I won’t get to be very old. (…) Will my husband be able to care for them financially?"* Female, grade II glioma

Often, patients mentioned that a proactive attitude and good self-management skills (e.g., being able to look up relevant information and ask specific questions) were necessary to find one’s way within the Dutch healthcare and social security system. As not all patients possessed such skills, or skills were affected by the consequences of the tumour and/or its treatment, proactive outreach in- and outside the hospital setting was appreciated.

*"If you end up in this situation [being diagnosed with a glioma], it would be helpful to have a clear overview of upcoming [work-related] procedures. That way, you know what to expect. In reality, you don’t know anything, and you keep getting confronted with new pieces of relevant information, without ever seeing the full picture."* Female, grade II glioma

##### Professionals

Most professionals mentioned that they lacked knowledge regarding Dutch laws and regulations concerning sick leave, work disability, and reintegration. Whereas these professionals typically referred patients elsewhere (e.g., to social workers or to professionals in the patient’s workplace), most felt unsure as to who they could best refer their patients to. Most interviewed professionals noted that they missed guidance from an independent, specialized occupational physician. Professionals mentioned needing tailored, nuanced occupational guidelines for providing work-related support to malignant glioma patients.

*"I would certainly like to know where I can (…) refer people to."* Clinical nurse specialist

## Discussion

### Main findings

Patients with a malignant glioma who participated in this study reported various tumour- and treatment-related symptoms (e.g., fatigue, aphasia, heightened sensitivity to stimuli, heightened emotional responsivity) that they found impacted their work ability to varying extents (e.g., invoking a sense of discomfort in colleagues, not being able to drive, not being able to perform certain job tasks). According to professionals, the impact of the aforementioned symptoms on work ability is primarily influenced by the malignant glioma’s location, rather than its grade, with frontal lobe gliomas being considered most negatively impactful. Furthermore, although the aforementioned symptoms can present somewhat similarly in patients with a malignant glioma and patients with non-CNS tumours, professionals stated that these symptoms likely have a different or combined main cause (i.e., acquired brain injury and/or cancer treatment) and a different course (i.e., symptoms typically worsen over time in patients with a malignant glioma, whereas they might improve in patients with non-CNS tumours), warranting timely and tailored work participation support.

Patients who participated in this study did not currently experience work participation support as tailored, nor did professionals feel well equipped to provide tailored work participation support to this patient group. To improve tailoring of this kind of support, professionals suggested utilizing periodic neuropsychological assessments, improving access to relevant medical data (i.e., for occupational physicians), and developing tailored occupational guidelines for providing work participation support to this patient group.

### Interpretation of findings

To start, some patients reported vague symptoms (e.g., (suspected) burnout) pre*-*diagnosis. This is in line with previous research showing that glioma patients can experience impairments in various cognitive domains (e.g., memory and attention [25, 26]) early on [27]. Furthermore, most patients reported symptoms such as fatigue and cognitive impairment post-diagnosis/treatment. These experiences are reflected in the literature, e.g., a systematic review by IJzerman-Korevaar and colleagues (2018), in which the most prevalent symptoms in glioma patients were found to be, amongst others, cognitive impairments (36%), aphasia (24%), motor deficits (21%), and fatigue (20%) [28]. Of the ten most prevalent symptoms found in this systematic review, eight were related to the CNS and thus specific to glioma patients [28]. Apart from heightened emotional responsivity, interviewed patients did not report substantial psychiatric symptoms (i.e., changes in personality, behaviour, mood), though these can be present in patients with a malignant glioma, specifically those with prefrontal or temporal lobe gliomas [29].

In some patients we interviewed, symptoms such as fatigue and concentration difficulties negatively impacted their self-reported ability to participate in paid work. These symptoms partly overlap with symptoms known to hinder work participation in the wider cancer patient population (e.g., [30–32]). For patients with a malignant glioma, it is known that fatigue is a negative predictor of RTW [33]. Regarding cognitive impairments, Tanzilli and colleagues (2022) found that approximately 68% of 130 malignant glioma patients had a cognitive deficit in at least one cognitive domain. Cognitive domains that were most affected included executive function, long-term memory, and short-term memory [34]: cognitive functions that are largely related to the frontal lobe and are typically required to engage in paid work. Regarding work participation, in a study by Collins and colleagues (2013), it was reported that work tasks requiring working memory (i.e., following the flow of events, remembering one’s train of thought whilst speaking, putting together materials for a task, task-shifting, and following written instructions) were experienced as more problematic by working brain tumour survivors (i.e., patients diagnosed with a glioma) than by a working non-cancer control group [13]. In the current study, professionals stated that the location of the glioma was likely more impactful than its grade, with frontal lobe gliomas being particularly negatively impactful on work ability. Considering the abovementioned studies, and the fact that most glioma’s (circa 40%) affect the frontal lobe [35], this finding is corroborated by the literature.

Some patients had an optimistic view of their ability to participate in paid work, whereas others viewed it as unfeasible. Unfeasibility of RTW or work retention was often related by patients to glioma-specific consequences (e.g., aphasia making client contact undesirable), lack of support from their supervisor and/or occupational physician (e.g., feeling pushed towards taking up full disability benefits), or lack of possible work adjustments (e.g., the work being unamenable to adaptation). Provided that work adjustments could be made, professionals were mostly supportive of patients with a malignant glioma participating in paid work. Recently, a study by Bursi and colleagues (2023) showed that, although some cognitive symptoms persist, full work resumption is possible in some patients with malignant glioma [36]. However, it is unclear whether work adjustments were made for those patients. In our study, differences in illness- and work-related expectations sometimes affected the relationship between patients and professionals. Differences in views and opinions on the work ability of patients with a malignant glioma could be due to some professionals’ limited experience with glioma patients [37]. However, it might also be that professionals and patients had different foci when communicating about the malignant glioma and its relation to work. That is, professionals might have focused on the (im)permanence of cognitive impairments and the incurability of the illness, whereas patients might have focused on maintaining a sense of normalcy through work. Although not mentioned in the interviews, communication about illness-related and work-related expectations might be additionally complicated due to impaired social cognition and limited illness understanding in some patients with a malignant glioma [29].

When communicating with the workplace, most patients were open about the malignant glioma diagnosis and its related consequences as they experienced them. However, patients struggled knowing what to objectively expect regarding their current and future work ability (as also reflected in, e.g., [27]), and expressed a need for proactive outreach by professionals prior to worsening of symptoms. This uncertainty, coupled with cognitive difficulties such as impaired speech, made communicating about the illness at work difficult for some. Difficulties in communicating about the impact of cancer on patients’ work ability in- and outside the healthcare setting are not unique to patients with a malignant glioma. These difficulties also exist in patients with non-CNS tumours (e.g., [19, 38–40]) and in patients with other types of acquired brain injury (e.g., [41]). However, as prior studies have reported that 60% of patients with a malignant glioma experience stable or worsening cognitive impairments over time [34], whereas others improve over longer periods of time [26], it would be beneficial to provide these patients with timely, tailored, and periodic work participation advice, based on, e.g., comprehensive neuropsychological assessments [36, 42, 43].

Further hindering communication about work participation, patients and professionals noted that the work environment displayed signs of discomfort and uncertainty relating to the malignant glioma diagnosis. As the tumour could cause, e.g., epileptic seizures and personality and behavioural changes, patients and professionals noted that it was difficult for supervisors and colleagues to depend on them as an equal colleague. Although patients with non-CNS tumours and patients with non-cancer-related traumatic brain injury can experience discomfort in supervisors and colleagues as well, it could be that the majority of them experiences empathy and support in the work environment (e.g., [41, 44]). Thus, the tumour’s location and its specific consequences could potentially exacerbate feelings of discomfort in colleagues of patients with a malignant glioma. Because of the experienced discomfort, patients and professionals recommended psychosocial support for supervisors and colleagues as well.

Although the glioma-specific consequences on work ability would warrant the implementation of tailored work-related support, patients and professionals agreed that professionals typically offered generic work-related support designed for patients with cancer. Professionals’ reasons for not tailoring work-related support to the malignant glioma at present were, amongst others, a lack of accessible information regarding the possible impact of malignant glioma on work ability, and difficulties obtaining consent to view medical data of patients. Prior research has shown that a tailored occupational rehabilitation program can result in improved work performance in glioma patients [45], and that rehabilitation therapy for glioma patients can positively impact their functional prognosis and quality of life [46]. Improving accessibility of nuanced information on the consequences of a malignant glioma on work ability appears to be needed for professionals to offer tailored work-related support for these patients.

Lastly, patients expressed a need to expand their self-management skills to navigate their way through the Dutch healthcare and social security system. Currently, work-related conversations do not appear to be systematically offered within the Dutch healthcare system [40], necessitating many patients to seek help themselves. It is possible that glioma-specific consequences, e.g., fatigue and cognitive difficulties make it even harder for this patient group to locate and access appropriate support.

### Strengths and limitations

To our knowledge, this is the first qualitative study on work-related experiences and unmet needs of patients with a malignant glioma and relevant professionals. Contrasting with other studies on work participation in cancer patients, the majority of our patient interviewees was male, and professionals were embedded in various disciplines. The latter can also be viewed as a limitation, as it cannot be deduced whether the results of this study are carried broadly by professionals within and across disciplines. In the future, we recommend interviewing neurologists and neuropsychologists to ask about *assessment* of functional capacities of glioma patients. Furthermore, as occupational therapists are typically the main point of contact when *planning* return to work in the Netherlands, we recommend interviewing them to ask about unmet needs in providing work-related guidance to glioma patients.

Next, some patients might have experienced impaired long-term memory [34], contributing to a possible recall bias. This is a particular concern as mean time post-diagnosis was around 4 years, and cognitive symptoms tend to worsen over time [34]. In hindsight, it would have been relevant to ask interviewees about glioma subtypes and locations, to make more concrete inferences from our results. Additionally, due to COVID-19 restrictions, most interviews were held via telephone or via video call software, which might have made it more challenging to interact with interviewees, especially patients (e.g., not being able to observe non-verbal language).

Finally, this study was performed within the Dutch healthcare and social security context. As systems differ vastly between countries, it is important that readers distinguish between the more general experiences of interviewees, and ones that are influenced by the Dutch healthcare and social security context. According to laws and regulations in the Netherlands, employees who are sick have 2 years to work on their re-integration. During this time, employees typically receive 100% of their salary in the first, and 70% of their salary in the second year of being sick-listed. The employer and employee are jointly responsible for working on the employee’s re-integration. If re-integration is not (fully) possible within 2 years, (partial) work disability benefits can be applied for. Prior qualitative research on cancer patients in the Netherlands has addressed the potential unsuitability of this 2-year legal timeframe in the event of cancer [47]. In the event of brain cancer or any (cancer) diagnosis with an unpredictable prognosis, this timeframe can be experienced as particularly unsuitable. However, the aforementioned salary continuation and opportunities for receiving (partial) work disability benefits provide employees in the Netherlands with income security that likely differs from the experiences of employees in other countries (e.g., the UK [27]), thereby influencing our results [47].

### Implications for research and practice

Regarding future research, we recommend (1) involving patients with a malignant glioma in the development of tailored interventions on RTW and work retention, (2) including patients with a malignant glioma in intervention research on RTW and work retention, and (3) looking deeper into the discomfort experienced by the work environment and developing ways to support the work environment and the patient in cases of acquired brain injury, including malignant glioma.

Regarding practice, we recommend (1) integrating comprehensive neuropsychological assessments pre-RTW and several months post-RTW in patients with a relatively good prognosis, to objectively and subjectively assess neuropsychological functioning in patients with a malignant glioma, on the basis whereof various professionals can provide tailored work-related guidance, taking into account known risk factors for (stable or worsening) cognitive impairment and known work tasks that might be challenging for this patient population (e.g., those requiring executive functioning, long-term memory, and short-term memory), (2) adapting/supplementing instruments and guidelines (e.g., existing Dutch guidelines for occupational and insurance physicians [48, 49]) for labour experts and occupational physicians to aid in assessing, e.g., work functioning, extent of work disability, and insurance claims in patients with a malignant glioma and other incurable progressive illnesses (see more specific recommendations in [29]), (3) developing tools to improve knowledge of and communication about work-related topics in healthcare (e.g., in neuro-oncology departments) and work professionals using, e.g., an adaptation of the MiLES tool [50], and (4) providing psychosocial support within the work environment to constructively and respectfully address the needs, wishes, and realities of the patient with the malignant glioma diagnosis and his/her colleagues. To illustrate the third recommendation, in the Netherlands, e-learnings about cancer and work have been developed for nurses (i.e., [51]).

## Conclusion

Glioma-specific consequences on patients’ work ability require tailored guidance within the healthcare system and work environment. When this guidance is given, some patients with a malignant glioma are able to participate in paid work meaningfully and successfully. What has become clear is that patients and professionals require clarity regarding the functional capacities of patients with a malignant glioma, and that, moreover, patients require room to discuss their goals, needs, and preferences regarding the continuation of their working lives. To aid various professionals in providing tailored work-related guidance, comprehensive neuropsychological assessments pre-RTW and several months post-RTW should be considered, and nuanced guidelines for labour experts and occupational physicians should be realized. Patients and professionals should periodically initiate transparent conversations about work-related needs and expectations, and patients and the work environment should receive psychosocial support to manage (communication about) the consequences of a malignant glioma at work.

## Supplementary Information

Below is the link to the electronic supplementary material.

Supplementary file1 (DOCX 24 KB)

## Data Availability

Interview transcripts are available in Dutch upon reasonable request.
