# Supplementary material for: Work-related experiences and unmet needs of patients with a malignant glioma and relevant professionals: the BrainWork study
Source: J Cancer Surviv. 2023 Oct 2;19(1):326–38. doi: 10.1007/s11764-023-01469-z (PMC11814035; doi:10.1007/s11764-023-01469-z)
Supplement: Supplementary file 1 — Supplementary file1 (DOCX 24 KB) [file 11764_2023_1469_MOESM1_ESM.docx]

**Table 1** COREQ checklist

| **No Item** | **Guide questions/description** | **Paragraph** |
| --- | --- | --- |
| **Domain 1: Research team and reflexivity** | | |
| *Personal Characteristics* | | |
| 1. Interviewer/facilitator | Which author/s conducted the interview or focus group? | Heading ‘Data collection’, §1 |
| 2. Credentials | What were the researcher’s credentials? E.g. PhD, MD | Heading ‘Data collection', §1 |
| 3. Occupation | What was their occupation at the time of the study? | Heading ‘Data collection', §1 |
| 4. Gender | Was the researcher male or female? | --, --, --, and -- are female. -- is male. Information on author gender was not included in the manuscript as it was not thought to be relevant. |
| 5. Experience and training | What experience or training did the researcher have? | Heading ‘Data collection', §1 |
| *Relationship with participants* | | |
| 6. Relationship established | Was a relationship established prior to study commencement? | Heading ‘Data collection’ §1 |
| 7. Participant knowledge of the interviewer | What did the participants know about the researcher? e.g. personal goals, reasons for doing the research | Heading ‘Data collection’ §1  All interviewees provided *informed* consent, meaning they were fully informed of reasons for conducting the study, study aims etc. |
| 8. Interviewer characteristics | What characteristics were reported about the interviewer/facilitator? e.g. Bias, assumptions, reasons and interests in the research topic | Heading ‘Data collection’ §1  Information on the professional background of interviewers is given. |
| **Domain 2: study design** | | |
| *Theoretical framework* | | |
| 9. Methodological orientation and Theory | What methodological orientation was stated to underpin the study? e.g. grounded theory, discourse analysis, ethnography, phenomenology, content analysis | Heading ‘Analysis' |
| *Participant selection* | | |
| 10. Sampling | How were participants selected? e.g. purposive, convenience, consecutive, snowball | Heading ‘Sample and recruitment’, §1 and §2 |
| 11. Method of approach | How were participants approached? e.g. face-to-face, telephone, mail, email | Heading ‘Sample and recruitment’, §1 and §2 |
| 12. Sample size | How many participants were in the study? | Heading ‘Results – Sample characteristics’, §1 and §2 |
| 13. Non-participation | How many people refused to participate or dropped out? Reasons? | Information is available for patients, Heading ‘Results – Sample characteristics’, §1  Reasons for non-participation in professionals were mainly due to lack of time. |
| *Setting* | | |
| 14. Setting of data collection | Where was the data collected? e.g. home, clinic, workplace | Heading ‘Sample and recruitment’, §1 and §2 |
| 15. Presence of non-participants | Was anyone else present besides the participants and researchers? | Heading ‘Data collection’, §1 and §2 |
| 16. Description of sample | What are the important characteristics of the sample? e.g. demographic data, date | Heading ‘Results – Demographics’, §1 and §2 |
| *Data collection* | | |
| 17. Interview guide | Were questions, prompts, guides provided by the authors? Was it pilot tested? | Heading ‘Data collection’, §2 and Table 1 |
| 18. Repeat interviews | Were repeat interviews carried out? If yes, how many? | Heading ‘Data collection’, §2 |
| 19. Audio/visual recording | Did the research use audio or visual recording to collect the data? | Heading ‘Data collection’, §2 |
| 20. Field notes | Were field notes made during and/or after the interview or focus group? | Heading ‘Data collection’, §1 |
| 21. Duration | What was the duration of the interviews or focus group? | Heading ‘Data collection’, §1 |
| 22. Data saturation | Was data saturation discussed? | Heading ‘Sample and recruitment’, §3 |
| 23. Transcripts returned | Were transcripts returned to participants for comment and/or correction? | Heading ‘Data collection’, §2 |
| **Domain 3: analysis and findings** | | |
| *Data analysis* | | |
| 24. Number of data coders | How many data coders coded the data? | Heading ‘Analysis’ |
| 25. Description of the coding tree | Did authors provide a description of the coding tree? | No, but available in Dutch upon reasonable request |
| 26. Derivation of themes | Were themes identified in advance or derived from the data? | Heading ‘Analysis’ |
| 27. Software | What software, if applicable, was used to manage the data? | Heading ‘Analysis’ |
| 28. Participant checking | Did participants provide feedback on the findings? | Heading ‘Analysis’ |
| *Reporting* | | |
| 29. Quotations presented | Were participant quotations presented to illustrate the themes / findings? Was each quotation identified? e.g. participant number | Heading ‘Analysis’ |
| 30. Data and findings consistent | Was there consistency between the data presented and the findings? | Themes were identified from the data. Coding tree is available in Dutch upon reasonable request. |
| 31. Clarity of major themes | Were major themes clearly presented in the findings? | Heading ‘Results' |
| 32. Clarity of minor themes | Is there a description of diverse cases or discussion of minor themes? | Heading ‘Results’ |
